# Three *TFL1* homologues regulate floral initiation in the biofuel plant *Jatropha curcas*

**DOI:** 10.1038/srep43090

**Published:** 2017-02-22

**Authors:** Chaoqiong Li, Qiantang Fu, Longjian Niu, Li Luo, Jianghua Chen, Zeng-Fu Xu

**Affiliations:** 1 Key Laboratory of Tropical Plant Resources and Sustainable Use, Xishuangbanna Tropical Botanical Garden, Chinese Academy of Sciences, Menglun, Yunnan 666303, China; 2 College of Life Science and Agronomy, Zhoukou Normal University, Zhoukou, Henan, 466001, China; 3 Key Laboratory of Gene Engineering of the Ministry of Education, and State Key Laboratory for Biocontrol, School of Life Sciences, Sun Yat-sen University, Guangzhou 510275, Guangdong, China

## Abstract

Recent research revealed that *TERMINAL FLOWER 1 (TFL1*) homologues are involved in the critical developmental process of floral initiation in several plant species. In this study, the functions of three putative *TFL1* homologues (*JcTFL1a, JcTFL1b* and *JcTFL1c*) in the biofuel plant *Jatropha curcas* were analysed using the transgenic approach. *JcTFL1b* and *JcTFL1c*, but not *JcTFL1a*, could complement the *TFL1* function and rescue early flowering and determinate inflorescence phenotype in *tfl1-14 Arabidopsis* mutant, thus suggesting that *JcTFL1b* and *JcTFL1c* may be homologues of *TFL1*. Transgenic *Jatropha* overexpressing *JcTFL1a, JcTFL1b* or *JcTFL1c* showed late flowering, whereas only *JcTFL1b* and *JcTFL1c* overexpression delayed flowering in transgenic *Arabidopsis. JcTFL1b-*RNAi transgenic *Jatropha* consistently exhibited moderately early flowering phenotype. *JcFT* and *JcAP1* were significantly downregulated in transgenic *Jatropha* overexpressing *JcTFL1a, JcTFL1b* or *JcTFL1c*, which suggested that the late flowering phenotype of these transgenic *Jatropha* may result from the repressed expression of *JcFT* and *JcAP1*. Our results indicate that these three *JcTFL1* genes play redundant roles in repressing flowering in *Jatropha*.

Floral initiation, a key developmental process in higher plant life, involves transition from vegetative to reproductive growth. It is controlled by genetic pathways that integrate environmental cues such as temperature and day length as well as endogenous signals such as hormones, regulation of genes and the developmental status of the plant^1^,^2^.

The proper timing of flowering is the most critical aspect to ensure reproductive success^3^. In the model plant *Arabidopsis thaliana*, complex genetic networks for flowering are mainly regulated by five major pathways: the photoperiod, vernalisation, gibberellin, autonomous and age pathways^4^,^5^,^6^. In *Arabidopsis*, the floral induction signals from these five major flowering pathways are transmitted via floral integrator genes, such as *FLOWERING LOCUS T (FT*), *SUPPRESSOR OF OVEREXPRESSION OF CONSTANS1 (SOC1*) and *FLOWERING LOCUS C (FLC*), to the floral meristem identity genes *LEAFY (LFY*) and *APETALA1 (AP1*) at the apical meristem^6^,^7^,^8^,^9^.

*FT* belongs to the *FT*/*TFL1* gene family, which is similar to the phosphatidyl ethanolamine-binding protein (PEBP) gene family^2^,^7^,^10^. The *FT*/*TFL1* gene family includes three major subfamilies—*FT*-like, *TERMINAL FLOWER 1 (TFL1*)-like and *MOTHER OF FT AND TFL1 (MFT*)-like—in the plant^2^,^10^. In *Arabidopsis*, FT and TFL1 act as key flowering regulators playing antagonistic roles in the flowering transition^2^,^11^,^12^.

FT promotes flowering initiation, whereas TFL1 acts as a repressor for floral initiation and maintains the inflorescence meristem through suppression of the expression of *LFY* and *AP1*^12^,^13^,^14^,^15^. The *Arabidopsis tfl1* mutants produce fewer leaves and flower earlier than wild-type (WT) plants and the inflorescence meristem converts to a terminal flower^12^. In contrast, overexpression of *TFL1* genes from *Arabidopsis* and other plant species results in a lengthening of the vegetative phase, increased secondary inflorescence production and a delayed flowering in transgenic *Arabidopsis*^16^,^17^,^18^,^19^,^20^.

Study of *TFL1* homologues in various plant species has revealed similar as well as distinctive functions. For instance, constitutive expression of *CsTFL1* in chrysanthemums resulted in extremely late flowering and prevented upregulation of floral meristem identity genes in shoot tips and leaves^3^. Ectopic expression of the maize *TFL1* homologues produced similar phenotypes, including delayed flowering and altered inflorescence architecture^21^. Moreover, the overexpression of two *TFL1*-like genes, *CorfloTFL1* and *CorcanTFL1*, cloned from *Cornus florida* and *C. canadensis*, extended vegetative growth and delayed flowering in transgenic *Arabidopsis*, indicating functional conservation of *TFL1* homologues in control of transition to flowering^22^.

Overexpression of a homologue of *TFL1 (StTFL1*) in transgenic potato resulted in a marked increase in tuber number in the transgenic plants compared to the wild-type plants, which suggested that *StTFL1* was involved in the regulation of tuberisation^23^. A 2-bp deletion in the coding region of the*TFL1* homologue was responsible for continuous flowering behaviour in woodland strawberry^24^. The transgenic apples and pears expressing antisense transcripts of apple *TFL1* homologue (*MdTFL1*) displayed early flowering phenotypes and extreme reduction of the juvenile phase^25^. These studies therefore suggest that *TFL1* homologues have unique features in flowering time and plant architecture in each plant species^18^,^26^.

*Jatropha curcas* (physic nut), which belongs to the Euphorbiaceae family, is widely recognised as a potential bioenergy crop in tropical and subtropical regions due to its high oil content seeds and its adaptability to marginal land^27^,^28^. Indeed, the oil content of *Jatropha* seeds ranges from 30 to 50% by weight, and its oil exhibits excellent physicochemical properties such as low acidity, good stability, low viscosity and good cold flow properties. Therefore it is suitable for biodiesel production and industrial applications^29^,^30^. However, unstable and poor flowering causes low seed yield in *Jatropha*^31^. *Jatropha* shows diverse flowering behaviour in different cultivation areas. Normally, *Jatropha* flowers during the rainy season (one or two flowering peaks), but in permanently humid regions, flowering occurs throughout the year^32^. Also, the harvest of *Jatropha* seeds is time-consuming, and the harvest efficiency is low because of its continuous flowering^33^.

Molecular breeding will be an effective genetic improvement method to modify the flowering behaviour of *Jatropha* to obtain high-yielding *Jatropha* cultivars. The function analysis of *JcTFL1* genes is required for the comprehensive understanding of the molecular mechanism of flowering in *Jatropha*, which would be helpful for the genetic modification and improvement of *Jatropha* with optimal flowering behaviour.

We previously isolated six members of the *FT*/*TFL1* gene family and analysed their expression patterns throughout the vegetative and reproductive developmental stages in *Jatropha*. Three *TFL1* homologues were identified and named *JcTFL1a, JcTFL1b* and *JcTFL1c*, respectively^34^. Also, we analysed the function of an *FT* orthologue (*JcFT*) in *Jatropha* and found that *JcFT* acts as a flowering promoter^35^.

Chua *et al*.^36^ isolated two *Jatropha TFL1* homologues, *JcTFL1-1* and *JcTFL1-2* corresponding to *JcTFL1a* and *JcTFL1b* and preliminarily analysed their functions by overexpressing them in transgenic *Arabidopsis* and *Jatropha*. In their research, overexpression of *JcTFL1-1* or *JcTFL1-2* resulted in earlier flowering in transgenic *Arabidopsis*, which was different from our findings. *JcTFL1-1* overexpression *Jatropha* showed early flowering and self-pruning phenotypes, and *JcTFL1-2* overexpression *Jatropha* showed multiple shoots phenotype^36^, which were not found in our transgenic *Jatropha*.

To elucidate the biological functions of three *JcTFL1* genes in the floral initiation of *Jatropha* using a transgenic approach, we overexpressed *JcTFL1* genes in transgenic *Arabidopsis* and *Jatropha*, and downregulated *JcTFL1b* in transgenic *Jatropha* in this study. Our results show *JcTFL1* genes play important roles in the regulation of flowering behaviour in *Jatropha*, which would be helpful to improve seed yield of *Jatropha*.

## Results

### Function analysis of *JcTFL1* genes in transgenic *Arabidopsis*

**Figure 1:**
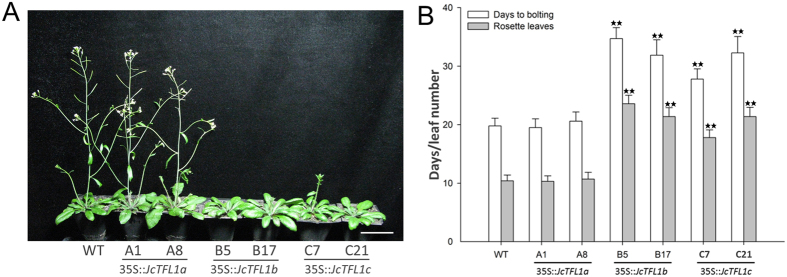
Flowering time of transgenic *Arabidopsis* (Col-0 background) with *JcTFL1* genes. (**A**) Growth of wild-type and *JcTFL1* overexpression transgenic lines in the Col-0 background under long-day (LD) conditions 40 days after germination. (**B**) Days and leaves to bolting for transgenic *Arabidopsis* lines in the Col-0 background and wild-type plants grown under LD conditions. Bar represents 5 cm. Values are means ± SD of the results from ten plants of each line. Student’s t-test was used to determine significant differences between the transgenic and WT plants. Significance level: **P < 0.01.

To determine the effects of *JcTFL1* genes on flowering time and inflorescence architecture, *JcTFL1a, JcTFL1b* and *JcTFL1c* cDNA driven by the constitutive 35S promoter were transformed into WT *Arabidopsis* plants, respectively. Consequently, we obtained >30 independent transgenic lines for 35S::*JcTFL1a*, 35S::*JcTFL1b* and 35S::*JcTFL1c*, respectively. For most 35S::*JcTFL1b* and 35S::*JcTFL1c* transgenic *Arabidopsis* lines, bolting occurred significantly later than in WT under long-day (LD) conditions (Fig. 1A,B). On the other hand, all transformants with 35S::*JcTFL1a* normally bolted like WT plants (Fig. 1A,B). Also, 35S::*JcTFL1b* and 35S::*JcTFL1c* transgenic plants had an increased rosette leaf number (Fig. 1A,B); in contrast, 35S::*JcTFL1a* transgenic *Arabidopsis* plants produced approximately the same rosette leaf number as WT plants (Fig. 1A,B). We also analysed expression levels of some flowering-related genes in WT and transgenic *Arabidopsis* by quantitative reverse transcriptase-polymerase chain reaction (qRT-PCR) experiment (Supplementary Fig. S2). Expression levels of all the flowering-related genes that we analysed was not significantly affected in 35S::*JcTFL1a* transgenic *Arabidopsis* lines (Supplementary Fig. S2D,H). On the other hand, *AtAP1* was significantly downregulated in the 35S::*JcTFL1b* and 35S::*JcTFL1c* transgenic lines (Supplementary Fig. S2E), which was consistent with the late flowering phenotype. However, the expression levels of flowering-related genes *AtLFY* and *AtSOC1* were not significantly affected in the transgenic lines (Supplementary Fig. S2F,G), which was not in accordance with the findings that *AtTFL1* repressed the expression of *AtLFY*^12^,^14^. We also analysed the expression levels of *AtTFL1*and *AtFT*, and unexpectedly found that the expression of *AtTFL1* and *AtFT* were downregulated in the 35S::*JcTFL1b* and 35S::*JcTFL1c* transgenic lines (Supplementary Fig. S2D,H).

**Figure 2:**
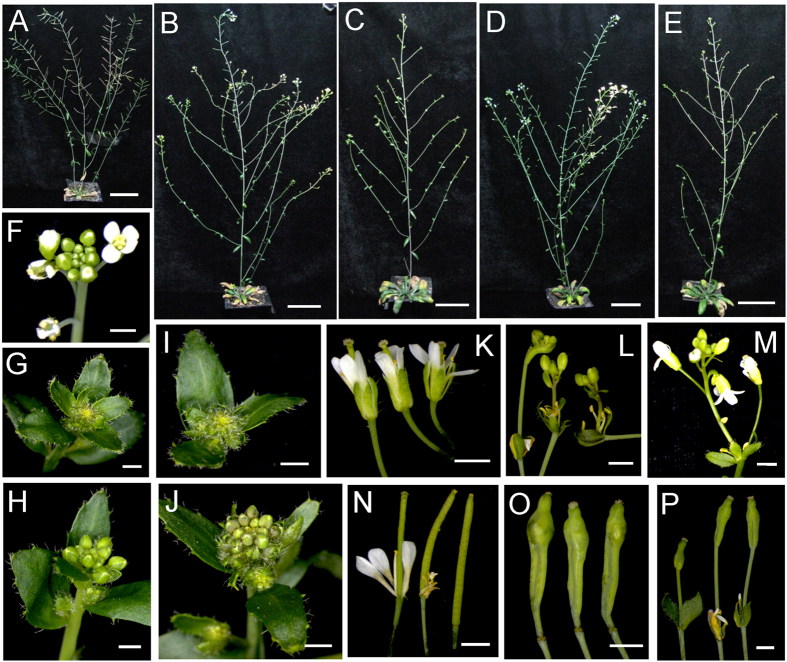
Plant architecture of transgenic *Arabidopsis* (Col-0 background) with *JcTFL1* genes. Whole plant (**A**), inflorescence (**F**), flowers (**K**) and siliques (**N**) of wild-type *Arabidopsis*; whole plant (**B** and **C**) and inflorescence buds (**G** and **H**) of 35S::*JcTFL1b* transgenic *Arabidopsis*; whole plant (**D** and **E**), inflorescence buds (I and J), inflorescences (**L** and **M**) and siliques (**O** and **P**) of 35S::*JcTFL1c* transgenic *Arabidopsis*. Bars in A–E represent 5 cm, and bars in F–P represent 1 mm.

We also found that more cauline branches were produced in the severe transgenic plants overexpressing *JcTFL1b* and *JcTFL1c* during the late development stage compared to WT plants (Fig. 2A–E), and 35S::*JcTFL1b* and 35S::*JcTFL1c* transgenic plants showed abnormality in the development of inflorescence, flowers and siliques. The shoot tips of severe transformants with 35S::*JcTFL1b* and 35S::*JcTFL1c* could not produce normal flower buds like WT plants, but instead a flower bud-like structure at the same position (Fig. 2F,G,I) which was similar to the phenotype overexpressing apple *TFL1* homologue in transgenic *Arabidopsis*^18^. Sometimes the tip consisted of small compact clusters of flower buds surrounded by leaves (Fig. 2H,J), and most of the flower buds could not develop into normal siliques. In addition, some unexpected phenotypes were observed in some of the transgenic *Arabidopsis* lines with 35S::*JcTFL1c* where a new inflorescence emerged from the position that should be pistil in WT plants (Fig. 2K,L,M), and the top portion of siliques in some 35S::*JcTFL1c* plants became abnormal (Fig. 2N,O,P). These data indicated that *JcTFL1b* and *JcTFL1c* might function as flowering repressors and *JcTFL1a* did not affect flowering time in transgenic *Arabidopsis*.

To investigate whether *JcTFL1* homologues function equivalently to *Arabidopsis TFL1*, we introduced the 35S::*JcTFL1a*, 35S::*JcTFL1b* and 35S::*JcTFL1c* vector into *Arabidopsis tfl1-14* mutant plants, which showed early flowering and determinate inflorescence phenotypes under LD conditions.

**Figure 3:**
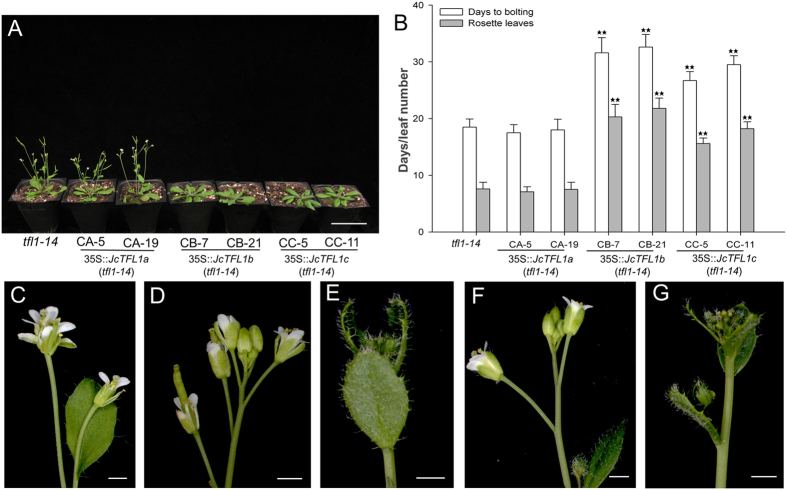
Overexpression of *JcTFL1* genes in transgenic *Arabidopsis (tfl1-14* background). (**A**) Growth of mutant *tfl1-14* and *JcTFL1* overexpression transgenic lines in the *tfl1-14* background under long-day (LD) conditions 23 days after germination. (**B**) Days and leaves to bolting for transgenic *Arabidopsis* lines in the *tfl1-14* background and mutant *tfl1-14* plants grown under LD conditions. (**C**) Inflorescence of mutant *tfl1-14*. (**D** and **E**) Inflorescences of transgenic *Arabidopsis* overexpressing *JcTFL1b*. (**F** and **G**) Inflorescences of transgenic *Arabidopsis* overexpressing *JcTFL1c*. Values are means ± SD of the results from ten plants of each line. Student’s t-test was used to determine significant differences between the transgenic and *tfl1-14* mutant plants. Significance level: **P < 0.01. Bar in A represents 5 cm, and bars in C-G represent 1 mm.

Consistent with the results overexpressing *JcTFL1* genes in WT *Arabidopsis* background, *tfl1-14* mutant plants transformed with 35S::*JcTFL1b* and 35S::*JcTFL1c* rescued the early flowering phenotype and maintained their indeterminate flowering behaviour similar to WT *Arabidopsis* plants (Fig. 3A–D,F; Supplementary Fig. S3). Moreover, some severe 35S::*JcTFL1b* and 35S::*JcTFL1c* transformants of *tfl1-14* background exhibited a similar phenotype in the shoot tip to these severe 35S::*JcTFL1b* and 35S::*JcTFL1c* transformants of WT background (Figs 2G–J,3E,3G).

On the other hand, overexpressing *JcTFL1a* could not rescue the early flowering and determinate inflorescence phenotype of *tfl1-14* mutant plants (Fig. 3A,B). These results supported the theory that *JcTFL1b* and *JcTFL1c* might be the orthologues of *TFL1* in *Jatropha* and that they affected flowering time and the inflorescence architecture in *Arabidopsis*.

Taken together, these findings demonstrated that ectopic expression of *JcTFL1b* and *JcTFL1c* in *Arabidopsis* delayed flowering and caused the changes in inflorescence architecture, while overexpression of *JcTFL1a* did not affect the flowering time and inflorescence architecture in transgenic *Arabidopsis*.

### Overexpression of *JcTFL1* genes affected flowering time and plant morphology in *Jatropha*

To determine the effects of *JcTFL1* genes on flowering time and plant morphology in *Jatropha*, we generated transgenic *Jatropha* plants with *JcTFL1a, JcTFL1b* and *JcTFL1c* under the control of the 35S promoter, respectively. Consistent with the results overexpressing *JcTFL1b* and *JcTFL1c* in *Arabidopsis*, transgenic *Jatropha* with 35S::*JcTFL1b* and 35S::*JcTFL1c* showed the extremely late flowering phenotype.

**Figure 4:**
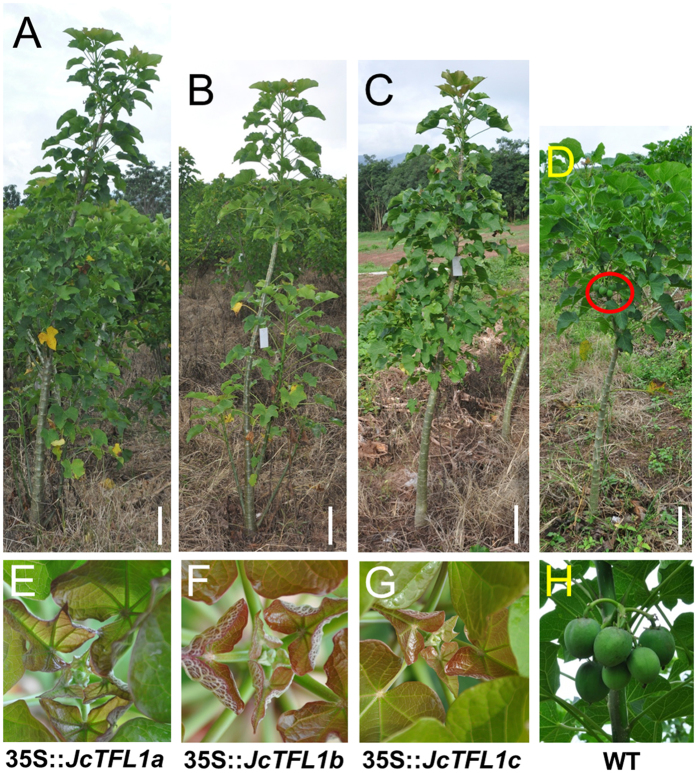
Overexpression of *JcTFL1* genes delay flowering in *Jatropha*. Transgenic *Jatropha* overexpressing *JcTFL1a* (**A**), *JcTFL1b* (**B**) and *JcTFL1c* (**C**) have not flowered yet 3 years after plantation in the field. Wild-type (WT) *Jatropha* (**D**) has set fruits about 10 months after plantation in the field; E, F and G correspond to the developmental stages of shoot apical of transgenic *Jatropha* shown in (**A**), (**B**) and (**C**), respectively. (**H**) The fruits of WT *Jatropha* shown in (**D**). The red circle indicates fruits. Bars represent 20 cm.

WT *Jatropha* flowered about 9 months after planting, and its height in the first flowering stage was about 1.5 m (Fig. 4D,H), while the 35S::*JcTFL1b* and 35S::*JcTFL1c* transgenic *Jatropha* flowered about 1.5 years after planting and reached a height of about 2.0 m. When we wrote this paper (3 years after planting), the extremely severe phenotype of 35S::*JcTFL1b* and 35S::*JcTFL1c* transgenic *Jatropha* had still not flowered (Fig. 4B,C,F,G).

Unexpectedly, 35S::*JcTFL1a* transgenic *Jatropha* also exhibited late flowering phenotype, which did not agree with the result in *Arabidopsis*—and the severest phenotype was similar to the 35S::*JcTFL1b* and 35S::*JcTFL1c* transgenic *Jatropha,* which had not flowered after 3 years (Fig. 4A,E). The heights of these transgenic plants are shown in Supplementary Fig. S4. Because of the no-flowering behaviour in these severe transgenic *Jatropha*, they grew no branches in the first year after transplanting to the soil (Supplementary Fig. S5). Meanwhile, WT *Jatropha* usually branched in the position where they flowered in the first year (Fig. 4D).

**Figure 5:**
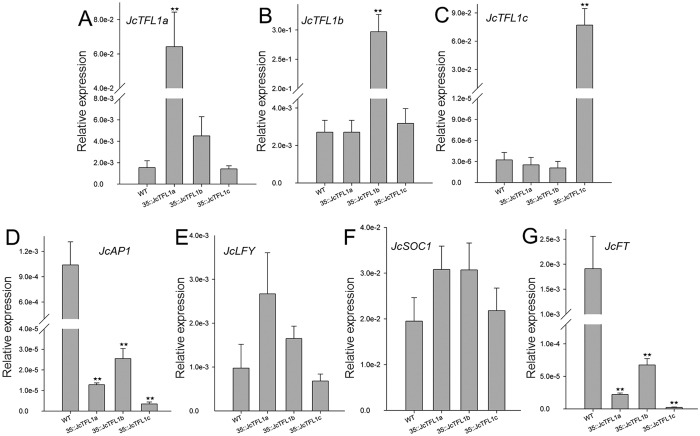
Quantitative RT-PCR analysis of several flowering-related genes in wild-type and transgenic *Jatropha* 2 years later after plantation in the field. (**A**) to (**G**) are expression levels of *JcTFL1a, JcTFL1b, JcTFL1c, JcAP1, JcLFY, JcSOC1*and *JcFT*, respectively. The qRT-PCR results were obtained from two independent biological replicates with three technical replicates each. Levels of the detected amplicons were normalized using the amplified products of *JcActin*.

To determine whether *JcTFL1* gene overexpression in the transgenic *Jatropha* changed the expression of some flowering-related genes—such as *AP1, LFY, FT* and *SOC1* homologues in *Jatropha*—qRT-PCR analysis was performed by using the shoot tip of the transgenic and WT *Jatropha* 2 years later after planting to soil (Fig. 5). Overexpression of each of three *JcTFL1* genes did not affect the transcripts of the other two *JcTFL1* genes (Fig. 5A–C).

As expected, *JcAP1* was significantly downregulated in three transgenic lines (Fig. 5D), which was consistent with the findings that TFL1 represses the expression of *AP1* in *Arabidopsis*^37^. However, the expression of *JcLFY* and *JcSOC1* was not apparently affected in these transgenic *Jatropha* (Fig. 5E,F), whereas *AtTFL1* repressed the expression levels of *AtLFY* in *Arabidopsis*^12^,^14^,^15^.

Meanwhile, we also detected the expression level of the florigen gene *JcFT* in transgenic *Jatropha* and found *JcFT* was significantly downregulated in these *JcTFL1* overexpressed transgenic *Jatropha* (Fig. 5G), which suggested that *JcTFL1* might repress the expression of *JcFT*.

### Silencing of the *JcTFL1b* gene moderately accelerated flowering in transgenic *Jatropha*

**Figure 6:**
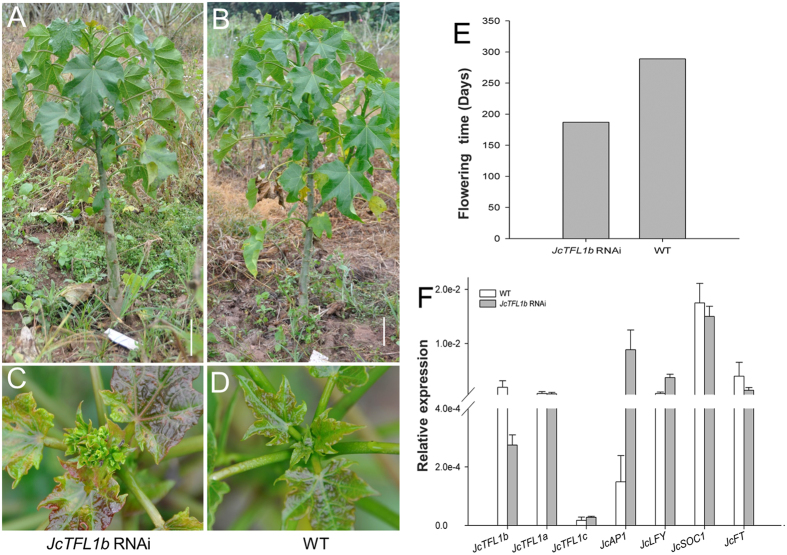
Silencing the expression of *JcTFL1b* moderately accelerated flowering in transgenic *Jatropha*. The whole plant (**A**) and flower buds (**C**) of *JcTFL1b* RNAi transgenic *Jatropha*. The whole plant (**B**) and leaf buds (**D**) of WT *Jatropha*. The photos were imaged about 6 months later after transplanting to soil. Bars represent 10 cm. (**E**) Flowering time of *JcTFL1b* RNAi transgenic *Jatropha*. Flowering time was scored by the number of days from transplantation to soil to the day of first inflorescence emergence. (**F**) Quantitative RT-PCR analysis of several flowering-related genes in wild-type and *JcTFL1b* RNAi transgenic *Jatropha*. The qRT-PCR results were obtained from two independent biological replicates with three technical replicates each. Levels of the detected amplicons were normalized using the amplified products of *JcActin*.

We previously found that among the three genes (*JcTFL1a, JcTFL1b*, and *JcTFL1c*) only *JcTFL1b* transcripts are abundantly accumulated in the flower buds of mature plants^34^. At the beginning of this study, we found that overexpression of *JcTFL1b* or *JcTFL1c*, but not *JcTFL1a*, delayed the flowering time in transgenic *Arabidopsis* plants (Fig. 1) and rescued the early flowering phenotype of *tfl1-14* mutant plants (Fig. 3). Therefore, we first chose *JcTFL1b* for further functional analysis by RNA interference (RNAi) in this study. We generated transgenic *Jatropha* containing the *JcTFL1b-*RNAi construct. These transgenic plants showed moderately early flowering, and produced flowers about 6 months later after planting to soil, which was 3 months earlier than that of WT *Jatropha* (Fig. 6E). The height of the first flowering *JcTFL1b* RNAi *Jatropha* was about 0.8 m (Fig. 6A,C), and the WT *Jatropha* remained as vegetative growth at the same time (Fig. 6B,D). In addition, we also detected the expression levels of some flowering-related genes in *JcTFL1b* RNAi *Jatropha* and found *JcAP1* was remarkably upregulated (Fig. 6F). These findings further demonstrated that *JcTFL1b* acts as a flowering repressor in *Jatropha*.

## Discussion

FT and TFL1 are thought to be molecular switches for vegetative growth to reproductive development in *Arabidopsis*. Moreover, FT promotes flowering and TFL1 represses flowering^2^,^6^,^22^.

In the present study, we analysed the function of three *JcTFL1* genes using the transgenic approach. We found that *JcTFL1b* and *JcTFL1c* could delay flowering in transgenic *Arabidopsis* and *Jatropha* and that both of them could also rescue early flowering and determinate inflorescence phenotype of *Arabidopsis tfl1-14* mutant (Figs 1,3,4; Supplementary Fig. S3). However, while *JcTFL1a* did not affect the flowering time in transgenic *Arabidopsis* in both WT and *tfl1-14* background, it could delay flowering in *Jatropha* (Figs 1,3,4), which might be the results of ectopic expression in *Arabidopsis*. In addition, *JcTFL1b* RNAi *Jatropha* showed moderately early flowering phenotype (Fig. 6), which was similar to *MdTFL1* RNAi apple that displayed early flowering phenotypes and extreme reduction of the juvenile phase^25^.

These findings suggested that three *JcTFL1* genes act as flowering repressors in *Jatropha* and that they might act redundantly in repressing flowering time. Chua *et al*. also analysed the functions of *JcTFL1-1*and *JcTFL1-2* corresponded to our *JcTFL1a* and *JcTFL1b*, respectively^36^. They found early flowering phenotypes in transgenic *Arabidopsis* plants overexpressing *JcTFL1-1* and *JcTFL1-2*, early flowering and self-pruning phenotypes in *JcTFL1-1* overexpression *Jatropha* and multiple shoots phenotype in *JcTFL1-2* overexpression *Jatropha*^36^. We did not find these phenotypes in our transgenic *Arabidopsis* and *Jatropha*. Chua *et al*. considered that JcTFL1-1and JcTFL1-2 also functioned as flowering activator or florigen like JcFT in *Jatropha*, which was totally opposite to our results. The function analysis of the *TFL1* homologues in apple^18^, chrysanthemum^3^, *Cornus florida* and *C. canadensis*^22^, gentian^38^, orchid^39^ and other plant species demonstrated that TFL1 homologues act as flowering repressors, which is consistent with our results.

The *JcTFL1* genes also affected the plant morphology in *Arabidopsis* and *Jatropha*. Transgenic *Arabidopsis* plants overexpressing *JcTFL1b* and *JcTFL1c* showed more cauline branches phenotype (Fig. 2A,E), but transgenic *Jatropha* plants overexpressing each of three *JcTFL1* genes showed no branches in the first year after planting (Supplementary Fig. S5), which may be the result of different plant species that generated new branches through different mechanisms. Transgenic *Arabidopsis* plants overexpressing *JcTFL1c* exhibited abnormal flowers and siliques (Fig. 2L–M,O–P), which suggested that *JcTFL1c* might have multiple functions in plant development.

To better understand the roles of *JcTFL1* genes, we detected the expression levels of some flowering-related genes in transgenic *Arabidopsis* and *Jatropha*, and found that the expression patterns of the flowering-related genes in transgenic *Arabidopsis* exhibiting late flowering were similar to that of transgenic *Jatropha. AtAP1*and *JcAP1* were significantly downregulated in overexpression transgenic *Arabidopsis* (Supplementary Fig. S2E) and *Jatropha* (Fig. 5D), which was consistent with the result that AtTFL1 represses the expression levels of *AtAP1* in *Arabidopsis*^3^,^37^. At the same time, *JcAP1* was remarkably upregulated in *JcTFL1b* RNAi transgenic *Jatropha* (Fig. 6F), which further demonstrated that JcTFL1b functioned as a flowering inhibitor in *Jatropha*. The expression levels of *AtLFY* were not affected in the transgenic *Arabidopsis* (Supplementary Fig. S2F), and the expression levels of *JcLFY* gene, the homologue of *LFY* in *Jatropha*, were also not obviously affected in transgenic *Jatropha* overexpressing *JcTFL1a, JcTFL1b*, or *JcTFL1c* and *JcTFL1b-*RNAi transgenic *Jatropha* (Figs 5E,6F), which was not agreed with the finding that TFL1 also repressed the expression levels of *LFY* in *Arabidopsis*^37^. In addition, the florigen gene *AtFT* and *JcFT* was significantly downregulated in transgenic *Arabidopsis* exhibiting late flowering phenotype (Supplementary Fig. S2H) and *Jatropha* overexpressing *JcTFL1a, JcTFL1b*, or *JcTFL1c* (Fig. 5G). Therefore, delayed flowering caused by overexpressing *JcTFL1* in transgenic *Arabidopsis* and *Jatropha* might result from the repressed expression of *AtAP1* and *AtFT, JcAP1* and *JcFT*, respectively.

## Materials and Methods

### Plant materials and growth conditions

Mature *Jatropha* seeds were collected from Xishuangbanna Tropical Botanical Garden of the Chinese Academy of Sciences, Mengla County, Yunnan Province, China. All control and transgenic *Jatropha* were also grown in Mengla County. WT *Arabidopsis thaliana* ecotype Columbia (Col-0), the *tfl1-14* mutant, and the transgenic lines were grown in peat soil in plant growth chambers at 22 ± 2 °C under a 16/8 h (light/dark) photoperiod, with cool-white fluorescent lamps used for lighting.

Transgenic *Arabidopsis* in the T_2_ or T_3_ generation were selected to examine flowering time and other phenotypes. For each genotype, 10 plants were used for characterisation; the number of leaves was counted along with the number of days between sowing and when the first flower bud was visible. All tissues prepared for qRT-PCR were immediately frozen in liquid nitrogen (N_2_) and stored at −80 °C until needed.

### Plant expression vector construction and *Arabidopsis* and *Jatropha* transformation

Total RNA was extracted from the shoot tip of *Jatropha* using the protocol described by Ding *et al*.^40^ First-strand cDNA was synthesised using M-MLV-reverse transcriptase from TAKARA (Dalian, China) according to the manufacturer’s instructions. The full-length *JcTFL1a, JcTFL1b* and *JcTFL1c* cDNA were obtained by PCR using the primer pairs *JcTFL1a* F and *JcTFL1a* R, *JcTFL1b* F and *JcTFL1b* R and *JcTFL1c* F and *JcTFL1c* R, which introduced *BamH* I and *Sac* I recognition sites, respectively. The PCR products were subsequently cloned into the pMD19-T and sequenced. Primers used in this study are listed in Supplementary Table S1.

To construct the plant overexpression vector 35S::*JcTFL1a*, the *JcTFL1a* sequence was excised from the pMD19-T simple vector using the restriction enzymes *BamH* I and *Sac* I and then cloned into the pBI121 vector containing the cauliflower mosaic virus 35S (35S) promoter. Plant overexpression vector 35S::*JcTFL1b* and 35S::*JcTFL1c* were also constructed following the steps described above. A *JcTFL1b* RNA interference (RNAi) vector was constructed for silencing the *JcTFL1b* transcripts. A 123-bp coding region of the *JcTFL1b* cDNA was selected and amplified by the PCR using the primers listed in Supplementary Table S1. The PCR products were cloned into a pJL10 vector^41^ in opposing orientations on either side of a pdk intron to create an invert repeat driven by the 35S promoter. A schematic representation of the plant transformation vectors used in this study is shown in Supplementary Fig. S1. The fidelity of the constructs was confirmed by PCR and restriction digestion.

Transformation of WT Col-0 and *tfl1-14* mutant plants with *Agrobacterium* strain EHA105 carrying the recombinant constructs was performed using the floral dip method^42^. Transgenic seedlings were selected for kanamycin resistance and confirmed by genomic PCR and RT-PCR.

Transformation of *Jatropha* with *Agrobacterium* strain LBA4404 carrying the overexpression construct was performed according to the protocol described by Fu *et al*.^43^. Transgenic *Jatropha* plants were confirmed by genomic PCR and RT-PCR.

### Expression analysis by qRT-PCR

*Arabidopsis* and *Jatropha* total RNAs were extracted from frozen tissue using TRIzol reagent (Transgene, China). Synthesis of the first-strand cDNA was performed following the methods described above. qRT-PCR was performed using SYBR^®^ Premix Ex Taq™ II (Takara Bio) on a Roche 480 Real-Time PCR Detection System (Roche Diagnostics).

The primers used for qRT-PCR are listed in lSupplementary Table S1. qRT-PCR was performed using two independent biological replicates and three technical replicates for each sample. Data were analysed using the 2^−ΔΔCT^ method as described by Livak and Schmittgen^44^. The transcript levels of specific genes were normalised using *Arabidopsis Actin2* or *Jatropha Actin*^45^.

## Additional Information

**How to cite this article:** Li, C. *et al*. Three *TFL1* homologues regulate floral initiation in the biofuel plant *Jatropha curcas. Sci. Rep.* **7**, 43090; doi: 10.1038/srep43090 (2017).

**Publisher's note:** Springer Nature remains neutral with regard to jurisdictional claims in published maps and institutional affiliations.

## Supplementary Material

Supplementary Figures and Table
