# Supplementary material for: Three TFL1 homologues regulate floral initiation in the biofuel plant Jatropha curcas
Source: Sci Rep. 2017 Feb 22;7:43090. doi: 10.1038/srep43090 (PMC5320528; doi:10.1038/srep43090)
Supplement: Supplementary Figures and Table [file srep43090-s1.pdf]

Three *TFL1* homologs regulate floral initiation in the biofuel plant *Jatropha curcas*

Chaoqiong Li[1,2,†], Qiantang Fu[1,†], Longjian Niu[1], Li Luo[3], Jianghua Chen[1], Zeng-Fu Xu[1,*]

Supplementary Materials

Table S1 Primers used in this study

Names	Sequences (from 5' to 3')
AtActin2 qRT-PCR F	TGT GCC AAT CTA CGA GGG TTT
AtActin2 qRT-PCR R	TTT CCC GCT CTG CTG TTG T
AtAP1 qRT-PCR F	GAC GTC AAT ACA AAC TGG TCG A
AtAP1 qRT-PCR R	GGA GAT GGC TGA TGA GAG AGC
AtFT qRT-PCR F	GAA CAA CCT TTG GCA ATG AGA
AtFT qRT-PCR R	TCT TCC TCC GCA GCC ACT
AtLFY qRT-PCR F	TCT CTC CCA AGA AGG GTT AT
AtLFY qRT-PCR R	GTA GTG TCG CAT TTT AGG CT
AtSOC1 qRT-PCR F	ACT AAA CGT AAA CTC TTG GGA
AtSOC1 qRT-PCR R	CAG AAC TTG GGC TAC TCT CT
AtTFL1 qRT-PCR F	ATA ATG GGG AGA GTG GTA GGA GA
AtTFL1 qRT-PCR R	TCT GGG TCT ATC ATC ACC AAA GT
JcActin qRT-PCR F	CTC CTC TCA ACC CCA AAG CCA A
JcActin qRT-PCR R	CAC CAG AAT CCA GCA CGA TAC CA
JcAP1 qRT-PCR F	GGG TTA TTT TGA GGA AAG AAG AGG A
JcAP1 qRT-PCR R	AAA CAA TCA AAG CAA CCT CAG CAT C
JcFT qRT-PCR F	AGG CAG ACC GTG TAT CCA CCA G
JcFT qRT-PCR R	ACT GAA TCA CCG TCT CCG TCC TC
JcLFY qRT-PCR F	GGA TAA GAT ACT ACA CAG CAG CGA
JcLFY qRT-PCR R	TAA CCC TTC TTG AGA GAG AGC ATC
JcSOC1 qRT-PCR F	TTC TTG GAC GGC AAC GCT TA
JcSOC1 qRT-PCR R	CTC TCG GAA AAG TGT GGG ATC
JcTFL1a qRT-PCR F	ACA GAC ATC CCA GGA ACA AC
JcTFL1a qRT-PCR R	AAG AAG ACA GCA GCA ACA GG
JcTFL1b qRT-PCR F	TCA CAA ATA CAG CAC CCA CC
JcTFL1b qRT-PCR R	CTT CTT GCA GCA GTT TCC CT
JcTFL1c qRT-PCR F	ACG GAG CCA CAG CCA CTT ACT GTA G
JcTFL1c qRT-PCR R	ACT CTA GGT TTA GCA GCA ATG ACC G
JcTFL1a F	GCG GAT CCT ATA TGG CAA AAG TGT CA
JcTFL1a R	CGG AGC TCT TAG CGT CTT CTT GC
JcTFL1b F	GCG GAT CCC AAT GGA AAA ACC AGT AG
JcTFL1b R	CGG AGC TCA AAT TAG CGT CTT CTT GC
JcTFL1c F	TAG GAT CCA TGT CAA GGG CGA CGG AG
JcTFL1c R	CGG AGC TCA TCA TCT TCT TCT GGC AGC
JcTFL1b RNAi-1	ACT CGA GAC AGA TGC CAC ATT TGG
JcTFL1b RNAi-2	TGG TAC CAG GTG GGT GCT GTA TTT G
JcTFL1b RNAi-3	CTC TAG AAC AGA TGC CAC ATT TGG
JcTFL1b RNAi-4	CAT CGA TAG GTG GGT GCT GTA TTT G

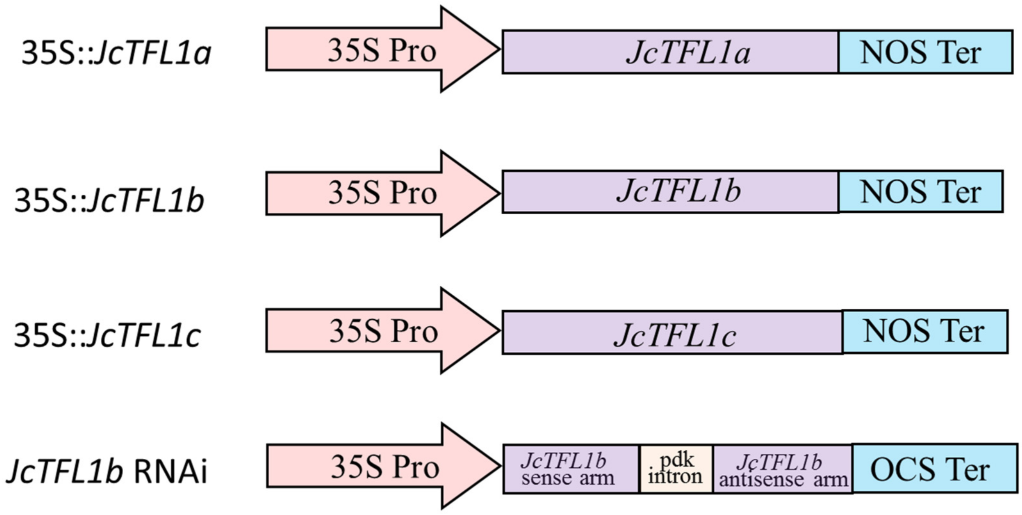

Supplementary Fig. S1 Schematic representation of the transformation vectors, 35S::*JcTFL1a*, 35S::*JcTFL1b*, and 35S::*JcTFL1c* used in this study. 35S Pro, cauliflower mosaic virus 35S promoter; NOS Ter, nopaline synthase gene terminator; OCS Ter, octopin synthase gene terminator.

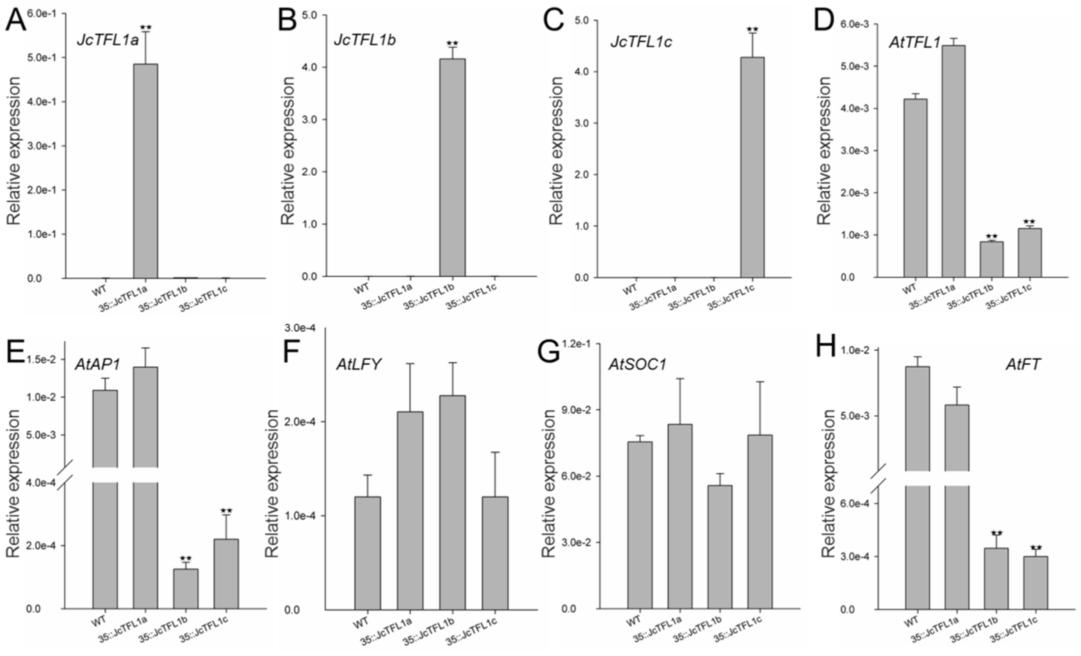

Supplementary Fig. S2 Quantitative RT-PCR analysis of several flowering-related genes in wild-type and transgenic *Arabidopsis*. (A) to (H) are expression levels of *JcTFL1a*, *JcTFL1b*, *JcTFL1c*, *AtTFL1*, *AtAP1*, *AtLFY*, *AtSOC1*and *AtFT*, respectively. *Arabidopsis* seedlings were collected 20 days after germination. The qRT-PCR results were obtained from two independent biological replicates with three technical replicates each. Levels of the detected amplicons were normalized using the amplified products of *AtActin2*.

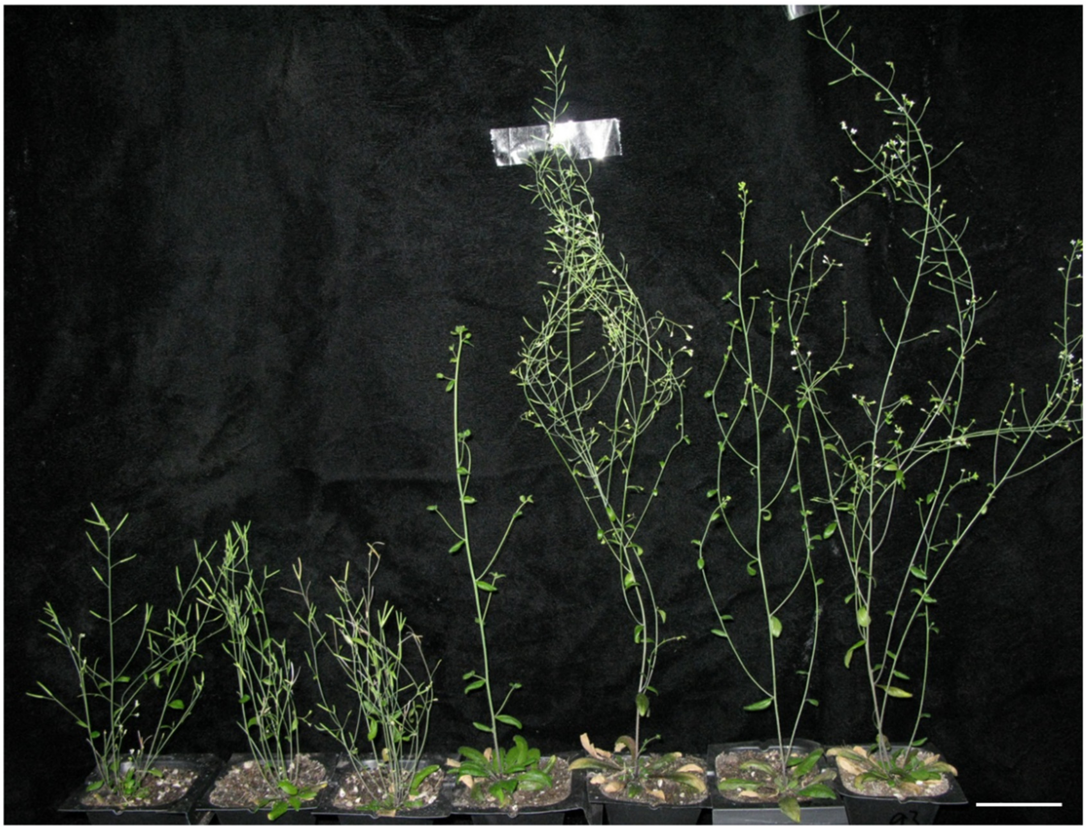

Supplementary Fig. S3 Overexpression of *JcTFL1*-like genes in transgenic *Arabidopsis* (*tfl1-14* background) under long-day conditions 55 days after germination. Bar represents 5 cm.

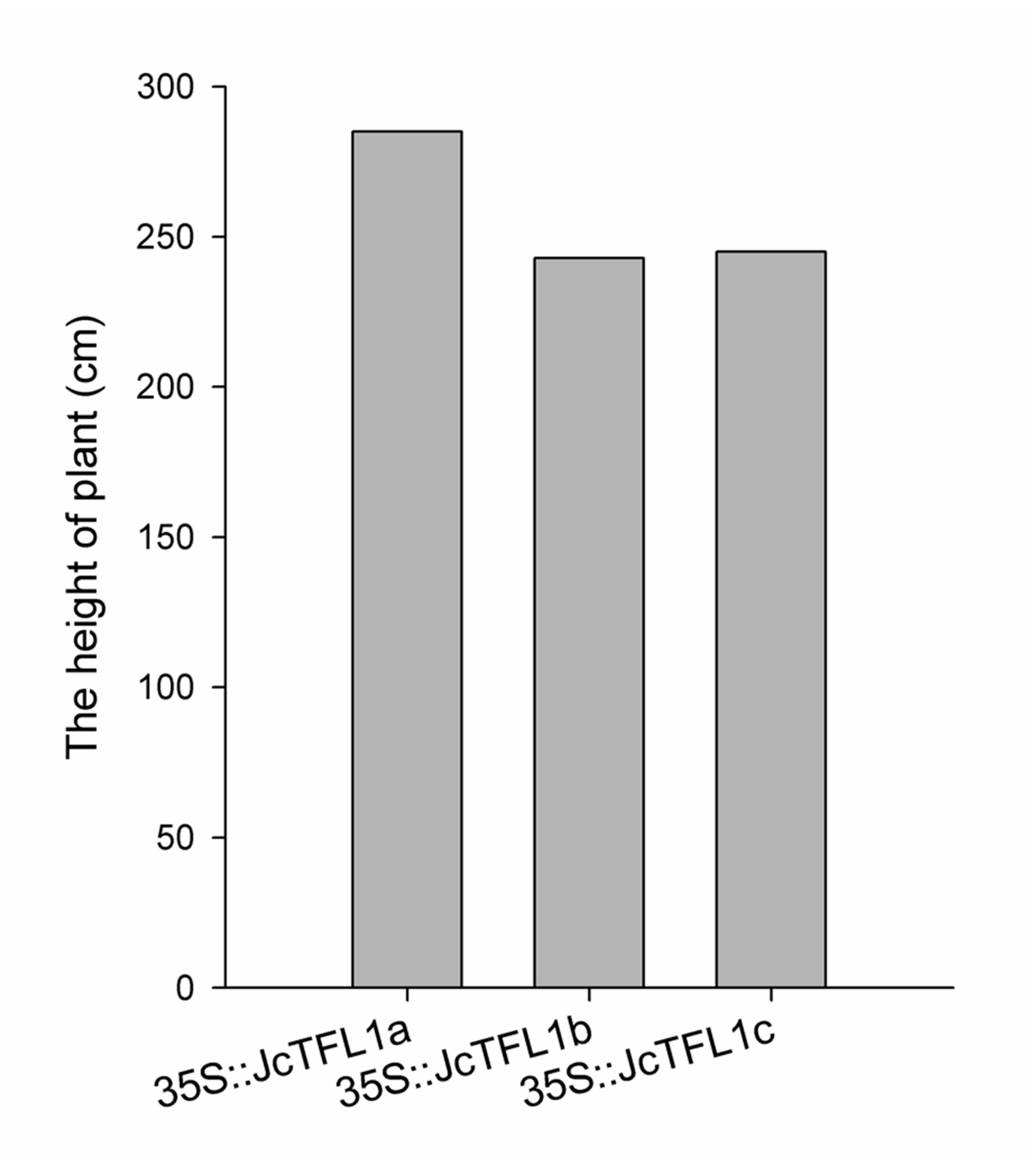

Supplementary Fig. S4   The height of transgenic *Jatropha* overexpressing *JcTFL1a*, *JcTFL1b* and *JcTFL1c* two years after transplanting to the filed.

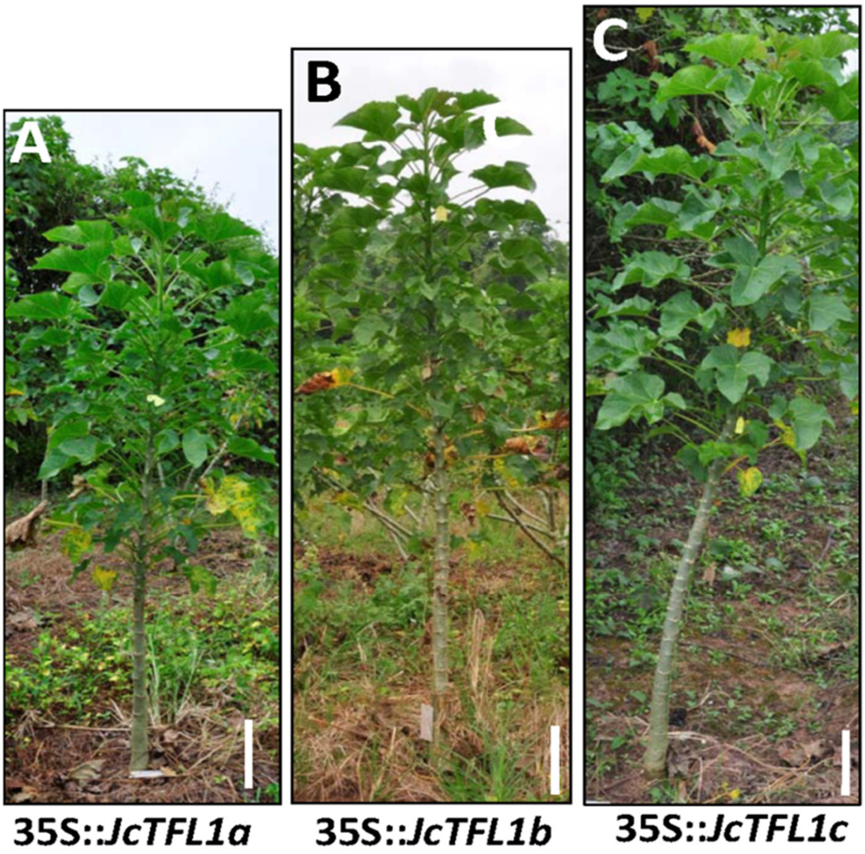

Supplementary Fig. S5 Overexpression of *JcTFL1* genes changed plant morphology in *Jatropha*. No branch was found in transgenic *Jatropha* overexpressing *JcTFL1a* (A), *JcTFL1b* (B) and *JcTFL1c* (C) in the first year after transplanting to the filed. Bars represent 20 cm.
